# A BCLC-based prognostic nomogram for hepatocellular carcinoma: Development and validation

**DOI:** 10.1016/j.isci.2026.115806

**Published:** 2026-04-17

**Authors:** Na Li, Di Sun, Xin-Cheng He, Bao-Hua Zhang, Ji Wang, Ming-Cheng Guan, Hong Zhu

**Affiliations:** 1 Department of Medical Oncology, The First Affiliated Hospital of Soochow University, Suzhou, Jiangsu, China; 2 Department of Oncology, The Second Affiliated Hospital of Soochow University, Suzhou, Jiangsu, China; 3 Department of Medical Oncology, Affiliated Hospital of Jiangnan University, Wuxi, Jiangsu, China

**Keywords:** Health sciences, Hepatology, Internal medicine, Medical specialty, Medicine

## Abstract

This study aimed to improve prognostic prediction for hepatocellular carcinoma by incorporating additional clinical factors into the Barcelona Clinic Liver Cancer staging system. A nomogram was developed using retrospective data from 536 patients and validated in an independent cohort of 102 patients. Independent prognostic factors identified by Cox regression included age, alpha-fetoprotein, gamma-glutamyl transpeptidase, and the Barcelona Clinic Liver Cancer stage. The nomogram showed superior discrimination and calibration compared with the staging system alone, with consistent results in the validation cohort. This tool enables more individualized survival estimation and may assist in clinical decision-making for patients with hepatocellular carcinoma.

## Highlights

• Combining age, AFP, rGT, and BCLC stage improves HCC prognostic prediction via a nomogram
• The nomogram shows better discrimination and net clinical benefit than BCLC staging alone
• Risk stratification using the nomogram effectively distinguishes overall survival outcomes

## Graphical abstract

**Figure:**
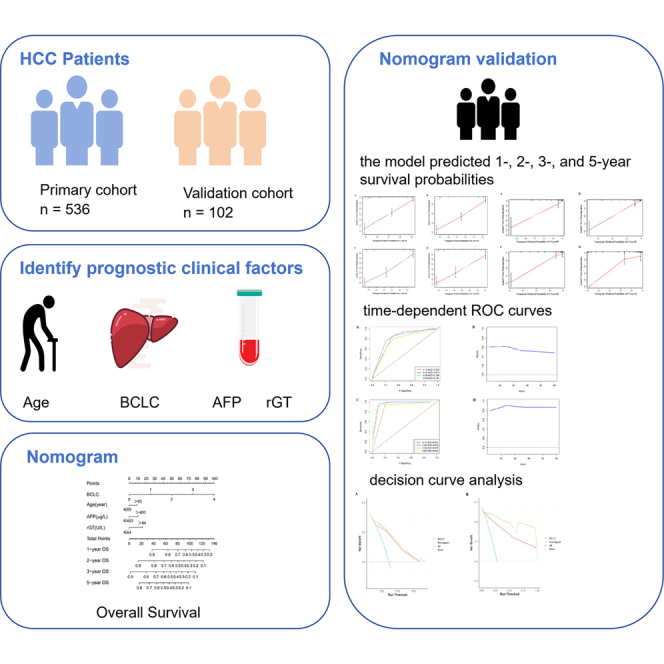

## Introduction

Hepatocellular carcinoma (HCC) ranks as the sixth most common malignancy globally and represents the third leading cause of cancer-related mortality.^1^ Owing to the lack of early clinical manifestations, most patients are diagnosed at advanced stages, rendering them ineligible for potentially curative treatments such as liver transplantation (LT) or hepatectomy. Consequently, the prognosis of HCC remains dismal, with a 5-year survival rate below 20% and a high recurrence rate. Although diagnostic and therapeutic strategies for HCC have continuously evolved, treatment outcomes vary substantially across disease stages. Therefore, precise staging is crucial for optimizing therapeutic decisions and predicting clinical outcomes.

Unlike many other malignancies, the prognosis of HCC is determined not only by tumor-related features—such as size, number, vascular invasion, and metastasis^2^—but also by the underlying hepatic functional reserve.^3^ Numerous clinical staging systems for HCC, including the American Joint Committee on Cancer (AJCC) TNM staging, Barcelona Clinic Liver Cancer (BCLC), Cancer of the Liver Italian Program (CLIP), and the Chinese University Prognostic Index (CUPI), have been developed and are widely used in clinical practice. Among them, the BCLC staging system,^4^ introduced in 1999, represented a major advance by integrating liver function, tumor characteristics, and patient performance status. It has since gained wide acceptance and is endorsed by leading organizations such as the European Association for the Study of the Liver (EASL), the European Organization for Research and Treatment of Cancer (EORTC), and the American Association for the Study of Liver Diseases (AASLD). Nevertheless, the BCLC staging system has undergone minimal revisions over the past decade,^5^^,^^6^^,^^7^ and its initial development was based predominantly on Western populations, raising concerns about its suitability for patients with Chinese HCC.

Gomaa et al. compared multiple HCC staging systems in an Egyptian cohort and reported that BCLC most effectively stratified patients by prognosis, whereas the CLIP score provided superior prognostic accuracy for advanced disease, highlighting the prognostic importance of including alpha-fetoprotein (AFP).^8^ Additionally, serum gamma-glutamyl transpeptidase (rGT) has emerged as a prognostic marker in several malignancies, including ovarian,^9^ renal,^10^ and esophageal cancers.^11^ Elevated rGT levels have also been associated with unfavorable outcomes following hepatectomy, radiofrequency ablation (RFA), and transcatheter arterial chemoembolization (TACE) in patients with HCC.^12^^,^^13^^,^^14^ In addition, the incidence of HCC in elderly individuals has been increasing,^15^ and advanced age is often linked to poorer survival due to frailty and comorbidities. Collectively, these findings suggest that integrating additional prognostic indicators into the BCLC system could enhance its predictive performance for HCC survival.

Nomograms are quantitative tools that provide individualized prognostic predictions and have been shown to outperform traditional staging systems in estimating cancer survival. Previous studies have proposed nomograms for postoperative or recurrence-free survival prediction in HCC; however, these models primarily incorporated basic clinicopathological variables and often neglected established staging frameworks.^16^^,^^17^^,^^18^^,^^19^ Therefore, this study aimed to develop an optimized prognostic nomogram that integrates the BCLC system with supplementary prognostic factors, thereby improving the precision of survival prediction in patients with HCC.

## Results

### Baseline characteristics

Table 1 summarized the baseline clinical characteristics of 638 patients with HCC across the primary and validation cohorts. The majority of patients were male, accounting for 85.4% in the primary cohort and 73.5% in the validation cohort. The mean age was comparable between the two groups. Most patients had liver cirrhosis, observed in 64.4% and 64.7% of the primary and validation cohorts. HBV infection was the overwhelmingly predominant etiology, accounting for 72% and 68.6% of all patients in the primary and validation cohorts, respectively. In the primary cohort, 4 patients (0.7%) had alcohol-associated liver disease, compared with 1 patient (1.0%) in the validation cohort. Vascular invasion was present in 25.9% of patients in the primary cohort and 17.6% in the validation cohort.

**Table 1:** Baseline of HCC patient characteristics

Characteristics	Primary cohort *n* = 536	Validation cohort *n* = 102
Age (years)		
≤65	364(67.9)	69(67.6)
>65	172(32.1)	33(32.4)
Gender		
Male	458(85.4)	75(73.5)
Female	78(14.6)	27(26.5)
Etiology		
no underlying liver disease	50(9.3)	13(12.7)
HBV infection	386(72.0)	70(68.6)
HCV infection	19(3.5)	3(2.9)
HBV and HCV co-infection	1(0.2)	0(0.0)
non-alcoholic fatty liver disease	0(0.0)	0(0.0)
alcohol-associated liver disease	4(0.7)	1(1.0)
autoimmune hepatitis	1(0.2)	0(0.0)
primary biliary cholangitis	1(0.2)	0(0.0)
schistosomiasis liver disease	33(6.2)	5(4.9)
cryptogenic cirrhosis	41(7.6)	10(9.8)
Cirrhosis		
yes	345(64.4)	66(64.7)
no	191(35.6)	36(35.3)
Portal hypertension		
yes	158(29.5)	38(37.3)
no	378(70.5)	64(62.7)
Child-Pugh classification		
A	415(77.4)	63(61.8)
B	101(18.8)	31(30.4)
C	20(3.7)	8(7.8)
Tumor number		
single	319(59.5)	52(51.0)
multiple	217(40.5)	50(49.0)
Vascular invasion		
yes	139(25.9)	18(17.6)
no	397(74.1)	84(82.4)
Distant metastasis		
yes	79(14.7)	12(11.8)
no	457(85.3)	90(88.2)
Tumor location		
left lobe	76(14.2)	21(20.6)
right lobe	304(56.7)	55(53.9)
left and right lobes	142(26.5)	25(24.5)
caudate Lobe	14(2.6)	1(1.0)
BCLC stage		
0	27(5.0)	7(6.9)
A	190(35.4)	39(38.2)
B	74(13.8)	18(17.6)
C	224(41.8)	27(26.5)
D	21(3.9)	11(10.8)
Treatment regimen		
hepatectomy	234(43.7)	24(23.5)
TACE	216(40.3)	60(58.8)
RFA	13(2.4)	4(3.9)
targeted therapy or immunotherapy	54(10.1)	8(7.8)
conservative treatment	19(3.5)	6(5.9)
AFP (μg/L)		
≤400	324(60.4)	71(69.6)
>400	212(39.6)	31(30.4)
PT(s)		
≤13	330(61.6)	20(19.6)
>13	206(38.4)	82(80.4)
TB(μmol/L)		
≤23	362(67.5)	77(75.5)
>23	174(32.5)	25(24.5)
ALB(g/L)		
≤40	312(58.2)	56(54.9)
>40	224(41.8)	46(45.1)
ALT(U/L)		
≤50	360(67.2)	71(69.6)
>50	176(32.8)	31(30.4)
rGT(U/L)		
≤64	187(34.9)	43(42.2)
>64	349(65.1)	59(57.8)
Cr(μmol/L)		
≤97	508(94.8)	98(96.1)
>97	28(5.2)	4(3.9)
CK(U/L)		
≤310	531(99.1)	93(91.2)
>310	5(0.9)	9(8.8)

HCC, hepatocellular carcinoma; HR, hazard ratio; CI, confidence interval; HBV, hepatitis B virus; HCV, hepatitis C virus; BCLC, Barcelona Clinic Liver Cancer; TACE, transcatheter arterial chemoembolization; RFA, radiofrequency ablation; AFP, alpha fetoprotein; PT, prothrombin activity; TB, total bilirubin; ALB, albumin; ALT, alanine transaminase; rGT, gamma-glutamyl transpeptidase; Cr, creatinine; CK, creatine kinase.

### Overall survival in the two cohorts

The median follow-up duration for all patients was 20.8 months, with 19.3 months for the primary cohort and 30.7 months for the validation cohort. In the primary cohort, the median OS was 37.6 months (range: 11.3–110.8 months), with 1-, 2-, 3-, and 5- year OS rates of 73.4%, 59.4%, 50.3%, and 41.9%, respectively. In contrast, the validation cohort showed higher OS rates of 79.4%, 74.1%, 71.0%, and 61.3% at 1, 2, 3, and 5 years, respectively.

### Independent prognostic factors in the primary cohort

Univariate analyses (Table S1) identified several factors significantly associated with OS, including age (*p* = 0.011), portal hypertension (*p* < 0.001), Child-Pugh classification (*p* < 0.001), vascular invasion (*p* < 0.001), distant metastasis (*p* < 0.001), tumor number (*p* < 0.001), tumor location (*p* = 0.025), AFP (*p* < 0.001), PT (*p* < 0.001), TB (*p* < 0.001), ALB (*p* < 0.001), rGT (*p* < 0.001), and the BCLC stage (*p* < 0.001). Because the BCLC staging system already integrates variables such as tumor characteristics, liver function, and portal hypertension, multivariable Cox regression analysis excluded those overlapping factors to avoid covariance.

In the multivariate model (Table 2), age (*p* = 0.047), AFP level (*p* = 0.029), rGT (*p* = 0.003), and BCLC stage (*p* < 0.001) emerged as independent predictors of OS. Tolerance values >0.1 and variance inflation factor (VIF) values <10 indicated no multicollinearity among these variables. Collectively, older age, advanced BCLC stage, elevated AFP, and increased rGT levels were all associated with poorer survival outcomes.

**Table 2:** Multivariable analysis of OS of HCC in the primary cohort

Characteristics	HR	95%CI	*p*-value
Age	1.300	1.003–1.685	0.047
Tumor location	1.103	0.914–1.331	0.306
BCLC	2.035	1.772–2.336	<0.001
AFP	1.331	1.029–1.721	0.029
rGT	1.551	1.157–2.081	0.003

HCC, hepatocellular carcinoma; OS, overall survival; AFP, alpha fetoprotein; BCLC, Barcelona Clinic Liver Cancer; rGT, gamma-glutamyl transpeptidase; HR, hazard ratio; CI, confidence interval.

### Predictive performance of the nomogram

**Figure 1:**
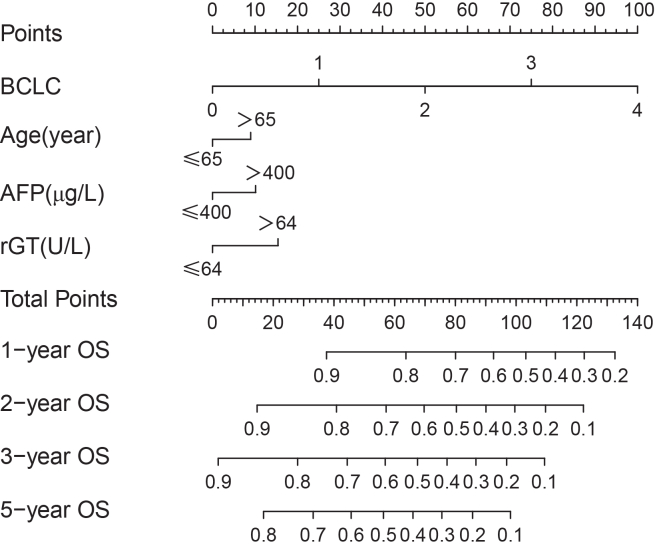
The nomogram was used to estimate the survival of patients with HCC To determine the OS probability, one draws vertical lines from the corresponding points accumulated for each factor. These points were then summed and aligned on a total points scale to predict survival rates at 1, 2, 3, and 5 years.

**Figure 2:**
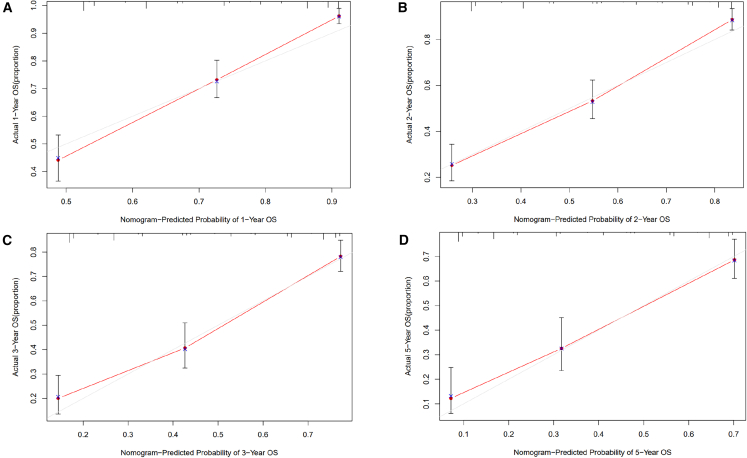
Comparison of observed versus nomogram-predicted OS rates at 1–5 years in the primary HCC cohort The actual observed 1-, 2-, 3-, and 5-year OS rates (A, B, C, D) were compared to the nomogram predictions in the primary cohort of patients with HCC.

A prognostic nomogram was constructed based on the independent predictors of OS identified in the primary cohort (Figure 1). The nomogram achieved a C-index of 0.750 (95% CI: 0.692–0.807), outperforming the BCLC staging system (C-index = 0.729, 95% CI: 0.678–0.780, *p* < 0.001), demonstrating superior discriminative power for predicting survival. Calibration plots indicated excellent concordance between predicted and observed survival probabilities at 1, 2, 3, and 5 years (Figure 2), confirming the model’s accuracy and stability.

### Validation of the nomogram

**Figure 3:**
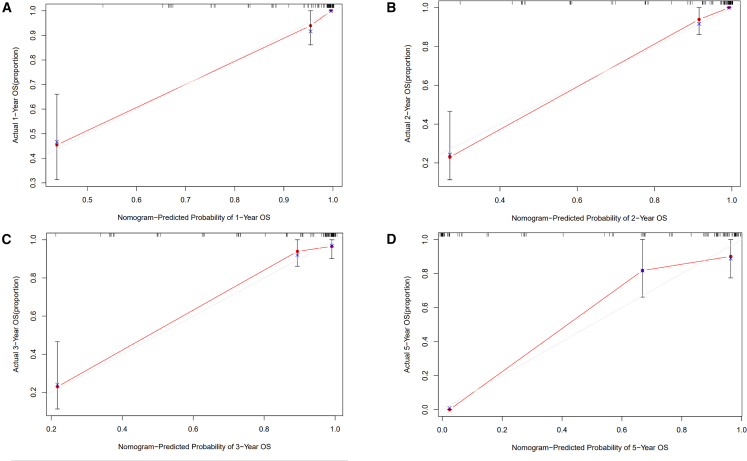
Comparison of observed versus nomogram-predicted OS rates at 1–5 years in the validation HCC cohort The observed 1-, 2-, 3-, and 5-year OS rates (A, B, C, D) in the validation cohort of patients with HCC were compared with the predictions generated by the nomogram.

When applied to the validation cohort, the nomogram maintained strong predictive accuracy despite variations in tumor characteristics. The C-index for OS prediction was 0.905 (95% CI: 0.824–0.986), exceeding that of the BCLC staging system (0.863, 95% CI: 0.779–0.947). The calibration curves at 1-, 2-, 3-, and 5-year time points demonstrated excellent alignment between predicted and actual survival outcomes (Figure 3).

### Time-dependent ROC analysis

**Figure 4:**
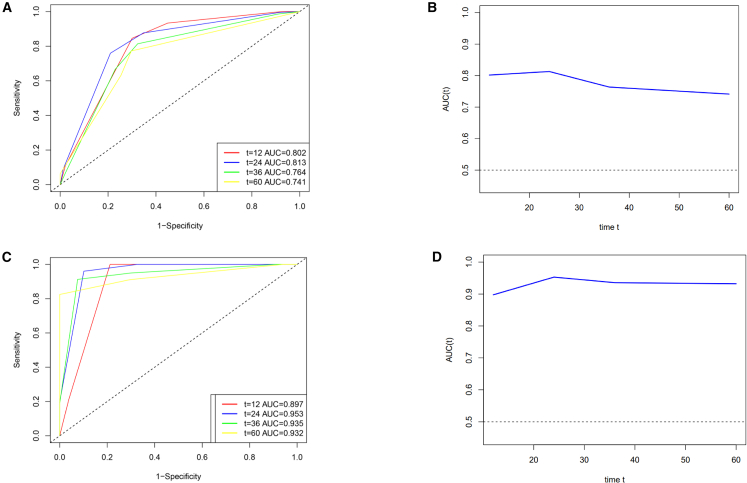
Time-dependent ROC curves and AUC values for the primary and validation cohorts The ROC curves for both the primary and validation cohorts over time (A, C) were presented, along with the corresponding AUC values over time (B, D).

Time-dependent ROC curves further validated the nomogram’s predictive performance (Figure 4). In the primary cohort, AUC values were 0.802, 0.813, 0.764, and 0.741 at 1, 2, 3, and 5 years, respectively (Figure 4A). Corresponding AUCs in the validation cohort were even higher—0.897, 0.953, 0.935, and 0.932 (Figure 4C). AUC values above 0.7 in the primary cohort (Figure 4B) and above 0.9 in the validation cohort (Figure 4D) indicated strong and consistent discriminatory capability.

### Comparison between the nomogram and BCLC staging system

Comparison using NRI and IDI analyses demonstrated that the nomogram provided greater prognostic accuracy than the BCLC system. In the primary cohort, NRI values for 2-year and 3-year OS were 0.156 (95% CI: 0.002–0.355, *p* < 0.05) and 0.138 (95% CI: 0.025–0.369, *p* < 0.05), respectively. Similar improvements were observed in the validation cohort. The nomogram also showed consistently positive IDI values for both 2-year and 3-year OS predictions (Table S2).

**Figure 5:**
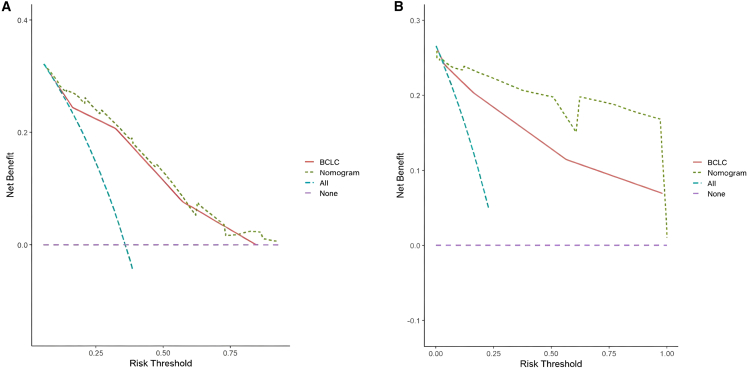
DCA was used to predict survival outcomes for patients with HCC, comparing the effectiveness of the nomogram with the BCLC staging system Panel (A) demonstrated the survival benefits in the primary cohort, while panel (B) illustrated the benefits in the validation cohort.

Decision curve analysis (Figure 5) further demonstrated that the nomogram provided a greater net clinical benefit than the BCLC staging system across both cohorts, underscoring its enhanced prognostic utility.

### Risk stratification based on the nomogram

**Figure 6:**
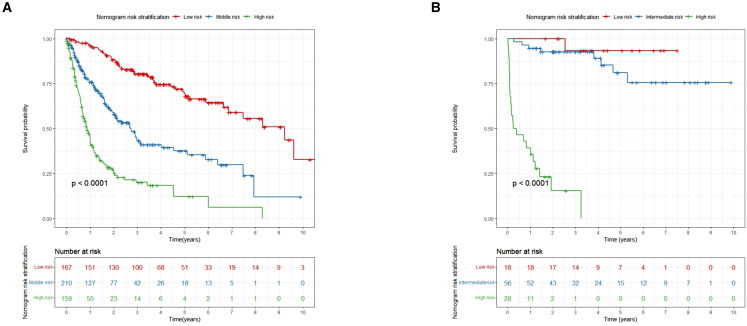
Kaplan-Meier overall survival curves of patients with HCC with different risks stratified by the nomogram (A) Patients with HCC in the primary cohort at different risks stratified according to the nomogram. (B) Patients with HCC in the validation cohort at different stages stratified according to the nomogram.

A risk stratification was finally performed based on the total points calculated using the nomogram. Patients were divided into three risk groups: low risk (total points <52.5), middle risk (52.5 ≤ total points <87.7), and high risk (total points ≥87.7). The Kaplan-Meier OS curves showed great discrimination among the three risk groups in both the training and validation cohorts (*p* < 0.001) (Figure 6).

## Discussion

Multiple staging systems have been proposed to predict prognosis and guide therapeutic decisions in HCC; however, no universal consensus has been reached regarding the optimal system for survival prediction or treatment stratification. The BCLC staging system, developed from large cohort data and randomized trials, has generally shown superior prognostic accuracy compared with other classification schemes. Previous studies demonstrated that the BCLC more effectively stratified prognosis for patients undergoing either surgical or non-surgical management.^20^^,^^21^^,^^22^^,^^23^ Its strength lies in integrating tumor burden, liver function, and patient performance status to guide treatment selection according to disease stage.

Nonetheless, certain limitations remain. The Child-Pugh classification within the BCLC system includes subjective parameters such as hepatic encephalopathy and ascites, which may introduce interobserver variability. Moreover, the system lacks molecular or biochemical indicators such as AFP that could refine prognostic assessment, particularly in early-stage HCC with solitary lesions.

To address these shortcomings, the present study established a nomogram incorporating three additional prognostic factors—age, AFP, and rGT—alongside the BCLC stage. All three variables have been validated in previous studies as significant determinants of HCC prognosis. Increasing age is associated with impaired hepatic regeneration, reduced portal perfusion, and decreased hepatic mass, leading to poorer clinical outcomes.^24^^,^^25^^,^^26^ Similarly, AFP, a well-known oncofetal protein expressed in approximately 70% of HCC cases,^27^^,^^28^ has been consistently linked to aggressive tumor biology, including vascular invasion and poor differentiation.^29^^,^^30^ Elevated AFP levels also predict unfavorable responses to liver transplantation, ablation, chemoembolization, and systemic therapy.^31^^,^^32^^,^^33^^,^^34^ Incorporation of AFP into existing staging systems, such as the Japan Integrated Staging (JIS) score, has improved prognostic precision in early-stage HCC.^35^ rGT, an enzyme involved in glutathione metabolism, plays a crucial role in oxidative stress regulation, inflammation, and tumor progression. Elevated serum rGT levels have been repeatedly associated with poor prognosis and reduced overall survival in patients with HCC.^36^^,^^37^^,^^38^^,^^39^^,^^40^^,^^41^^,^^42^ Therefore, combining these biomarkers with BCLC parameters offers a more comprehensive assessment of tumor burden and host condition.

In this study, the integrated nomogram demonstrated superior predictive performance, with a C-index of 0.750 (95% CI: 0.692–0.807), outperforming the BCLC staging system (0.729, 95% CI: 0.678–0.780, *p* < 0.001). Consistently positive NRI and IDI values further confirmed its discriminatory advantage. Calibration and decision curve analyses also verified that the nomogram yielded higher predictive accuracy and greater net clinical benefit compared with BCLC alone. The nomogram effectively stratified patients into three prognostic groups with markedly distinct overall survival outcomes in both cohorts. This consistent discriminative performance supports the generalizability of the model and underscores its clinical utility as a tool for individualized prognostic assessment and risk-adapted therapeutic strategies in HCC.

In summary, this study established a novel nomogram integrating age, AFP, and rGT with the BCLC staging system to refine survival prediction for patients with HCC. The nomogram exhibited superior predictive accuracy and clinical utility compared with BCLC alone, representing a potentially valuable tool for individualized prognosis assessment and treatment planning in HCC.

### Limitations of the study

Several limitations should be acknowledged. The retrospective design introduces inherent selection bias. Of note, OS in HCC represents a composite endpoint shaped by both tumor progression and underlying liver function. Reliance on OS alone limits the ability to discern whether prognostic factors capture tumor biology or hepatic functional reserve. Although recurrence-free or cancer-specific survival would provide more direct biological insights, such data were not uniformly obtainable in this retrospective cohort. Prospective follow-up with the systematic collection of recurrence events and cause-of-death data is planned. Subsequent application of competing risk models will enable differentiation between HCC-related and liver failure-related mortality, thereby clarifying the independent prognostic role of each variable. Moreover, as the cohort primarily comprised patients with HBV-related HCC from a single geographic region, the findings may not be fully generalizable to populations with different etiologies, such as HCV- or alcohol-related HCC. Therefore, multicenter prospective validation is warranted to confirm the model’s robustness and clinical applicability across diverse patient populations. Furthermore, as a real-world study spanning a period of evolving treatment paradigms, subsequent analyses will be stratified by both the primary treatment modality (e.g., curative-intent treatment group [hepatectomy], locoregional therapy group [TACE], and systemic therapy group) and the treatment era (such as 2010–2016 vs. 2017–2021). This will allow for comparison of whether the established nomogram maintains predictive performance across different treatment modalities and across distinct time periods. Finally, future studies are planned to include additional patients receiving contemporary immunotherapy-based regimens, thereby allowing further validation of the nomogram’s prognostic utility in this specific and increasingly relevant population.

## Resource availability

### Lead contact

Requests for further information and resources should be directed to and will be fulfilled by the lead contact, Hong Zhu (zhuhong_jasmine@suda.edu.cn).

### Materials availability

This study did not generate new unique reagents.

### Data and code availability

• All data reported in this paper will be shared by the lead contact upon request.
• This paper does not report original code.
• Any additional information required to reanalyze the data reported in this paper is available from the lead contact upon request.

## Acknowledgments

This study was supported by the 10.13039/501100001809National Natural Science Foundation of China (82171834, 82573671), 10.13039/501100002949Jiangsu Province Seventh 333 High Level Talents Project, Key Research and Development Program of Jiangsu Province (BE2022725), Beijing 10.13039/100016966Bethune Charitable Foundation (2023-YJ-119-J-023, 2023-YJ-042-S-014), Suzhou Science and 10.13039/100006180Technology Development Plan Project (SKY2023049), Research Project of Wuxi Municipal Health Commission (Q202523), and Provincial-level talent program for National Center of Technology Innovation for Bio Pharmaceuticals (NCTIB2024JS0101).

## Author contributions

N.L. and M.C.G. made the search strategy and searched the literature. D.S. and J.W. collected and extracted data. N.L. and X.C.H. provided software and performed data analyses. X.C.H. and B.H.Z. conducted quality assessment. N.L. drafted the initial manuscript. N.L., D.S., and B.H.Z. drew the figures. H.Z. and M.C.G. conceived the idea and revised the manuscript critically. All authors reviewed this manuscript and approved this submission.

## Declaration of interests

The authors declare no competing interests.

## STAR★Methods

### Key resources table

**Table:** 

REAGENT or RESOURCE	SOURCE	IDENTIFIER
**Software and algorithms**		

R software (4.2.2)	Open source	https://www.r-project.org/
R Studio	Open source	https://www.rstudio.com/
SPSS (Version 26.0)	IBM	https://www.ibm.com/
X-tile	Open source	http://www.tissuearray.org/soft/
rms R package (v4.2.2)	R CRAN	https://cran.r-project.org/web/packages/rms/index.html/
survminer R package (v4.2.2)	R CRAN	https://cran.r-project.org/web/packages/survminer/index.html/
ggplot2 R package (v4.2.2)	R CRAN	https://cran.r-project.org/web/packages/ggplot2/index.html/

### Experimental model and study participant details

#### Study approval and ethical statement

Ethics Approval and Patient Consent Statement: The study adhered to the principles of the 1975 Declaration of Helsinki and was approved by the Ethics Committees of the First and Second Affiliated Hospitals of Soochow University (2022–001). Informed consent was waived due to the retrospective nature of the study.

#### Study population

This retrospective study was conducted at the First and Second Affiliated Hospitals of Soochow University between January 2010 and December 2021. Consequently, 638 patients met the inclusion criteria and were divided into two cohorts: a primary cohort (*n* = 536) for model development and a validation cohort (*n* = 102) for external validation.

#### Inclusion and exclusion criteria

All enrolled patients had a confirmed diagnosis of HCC based on imaging modalities—including ultrasonography, computed tomography (CT), and magnetic resonance imaging (MRI)—and further verified through biopsy or postoperative histopathological examination. Initially, 1,459 consecutive HCC patients were identified. After applying exclusion criteria, 821 patients were excluded due to one or more of the following reasons: (1) age <18 years, (2) presence of another malignancy, (3) receipt of any anticancer treatment before enrollment, or (4) incomplete clinical data or follow-up information.

### Method details

#### Evaluation criteria

Comprehensive clinical and laboratory data were collected for all eligible patients. Demographic and clinical parameters included sex, age, etiology, and the presence of liver cirrhosis. The diagnosis of alcohol-associated liver disease was based on clinical practice guidelines, defined as HCC cases confirmed in patients with a history of long-term excessive alcohol consumption (men >60 g/day, women >40 g/day) after excluding other major liver disease etiologies, particularly viral hepatitis. Laboratory measurements comprised AFP, alanine aminotransferase (ALT), rGT, prothrombin time (PT), total bilirubin (TB), albumin (ALB), creatinine (Cr), and creatine kinase (CK). Tumor-related characteristics—such as the number and location of tumors, presence of portal hypertension, Child–Pugh classification, vascular invasion, and distant metastasis—were also assessed. For continuous laboratory variables, dichotomization was performed based on clinically established or laboratory-derived thresholds. The AFP cut-off of 400 μg/L was selected according to widely adopted clinical guidelines for hepatocellular carcinoma diagnosis and prognosis.^43^^,^^44^^,^^45^^,^^46^^,^^47^ All other laboratory parameters (ALT, rGT, PT, TB, ALB, Cr, CK) were dichotomized using the upper limit of the institutional normal reference range. All laboratory evaluations were performed within seven days before treatment initiation, while imaging assessments were completed within two weeks prior to therapy.

#### Study endpoints and follow-up

The primary endpoint of this study was overall survival (OS), defined as the time from the initial diagnosis of HCC to death from any cause. The final follow-up date was July 31, 2022.

### Quantification and statistical analysis

#### Development of the prognostic nomogram

A prognostic nomogram was developed using multivariable analyses of OS in the primary cohort. Variables such as tumor number, portal hypertension, Child-Pugh class, vascular invasion, distant metastasis, PT, ALB, and TB were excluded from the final multivariable analysis and nomogram, as these parameters are already encompassed within the BCLC staging system. The final model was established using a backward stepwise selection approach. Model discrimination was quantified using the concordance index (C-index), with values ranging from 0.5 (no discrimination) to 1.0 (perfect discrimination). Kaplan–Meier survival curves were generated to visualize survival probabilities, and calibration plots were applied to evaluate the agreement between predicted and observed outcomes. Internal validation was conducted using 1,000 bootstrap resamples.

Categorical variables were expressed as proportions, and continuous variables were summarized as means or medians, as appropriate. Multivariable Cox proportional hazards regression models were applied to identify independent prognostic factors, and results were visualized as a nomogram using the rms package in R (version 4.2.2; http://www.r-project.org/). The model predicted 1-, 2-, 3-, and 5-year survival probabilities for individual HCC patients. Both internal and external validation were performed via the bootstrap method, and the predictive performance was evaluated using the C-index and calibration curves. Time-dependent receiver operating characteristic (ROC) curves were generated to assess discriminatory power further. Comparative analyses between the nomogram and the BCLC staging system were conducted using C-index, net reclassification improvement (NRI), integrated discrimination improvement (IDI), and decision curve analysis (DCA), with corresponding *p*-values. Risk stratifications with the nomogram were compared using the Kaplan-Meier method and the Cox model. The cut-off point for risk stratification was selected using X-tile.^48^ All statistical analyses were performed using IBM SPSS Statistics version 26.0 (SPSS Inc., Chicago, IL, USA) and R software. A two-tailed *p* value <0.05 was considered statistically significant.
