# Supplementary material for: A BCLC-based prognostic nomogram for hepatocellular carcinoma: Development and validation
Source: iScience. 2026 Apr 17;29(5):115806. doi: 10.1016/j.isci.2026.115806 (PMC13157075; doi:10.1016/j.isci.2026.115806)
Supplement: Document S1. Tables S1 and S2 [file mmc1.pdf]

iScience, Volume *29*

Supplemental information

A BCLC-based prognostic nomogram for hepatocellular carcinoma: Development and validation

Na Li, Di Sun, Xin-Cheng He, Bao-Hua Zhang, Ji Wang, Ming-Cheng Guan, and Hong Zhu

Supplementary Table 1. Univariate Analysis of OS of HCC in primary cohort.

Characteristics	HR	95%CI	*P*-value
**Gender**	1.222	0.871-1.714	0.246
**Age**	1.395	1.080-1.800	0.011
**Cirrhosis**	0.955	0.738-1.236	0.726
**portal hypertension**	1.634	1.265-2.112	＜0.001
**Child-Pugh classification**	1.803	1.453-2.238	＜0.001
**vascular invasion**	3.374	2.582-4.409	＜0.001
**distant metastasis**	5.619	4.085-7.730	＜0.001
**tumor number**	2.224	1.727-2.863	＜0.001
**tumor location**	1.219	1.025-1.449	0.025
**BCLC**	2.192	1.926-2.494	＜0.001
**AFP**	1.671	1.301-2.148	＜0.001
**PT**	1.576	1.229-2.020	＜0.001
**TB**	1.577	1.227-2.027	＜0.001
**ALB**	1.712	1.326-2.211	＜0.001
**ALT**	1.082	0.833-1.406	0.554
**rGT**	2.331	1.753-3.098	＜0.001
**Cr**	1.587	0.982-2.564	0.059
**CK**	1.781	0.662-4.794	0.253

OS, overall survival; HCC, hepatocellular carcinoma; BCLC, Barcelona Clinic Liver Cancer; AFP, alpha fetoprotein; TB, total bilirubin; PT, prothrombin activity; ALT, alanine transaminase; rGT, gamma-glutamyl transpeptidase; ALB, albumin; Cr, creatinine; CK, Creatine Kinase; HR, hazard ratio; CI, confidence interval.

Supplementary Table 2. Comparison of nomogram with BCLC by IDI to predict prognostic ability

		**IDI（95%CI）**	***P*-value**
**Primary cohort**	2year OS	0.023（0.003 - 0.056）	0.030
	3year OS	0.024（0.006 - 0.057）	0.008
**Validation cohort**	2year OS	0.214（0.089 - 0.352）	0.006
	3year OS	0.185（0.048 - 0.322）	0.016

OS, overall survival; BCLC, Barcelona Clinic Liver Cancer; IDI, Integrated discrimination improvement; CI, confidence interval.
